# Foliar Application of Ethylenediamine Tetraacetic Acid (EDTA) Improves the Growth and Yield of Brown Mustard (*Brassica juncea*) by Modulating Photosynthetic Pigments, Antioxidant Defense, and Osmolyte Production under Lead (Pb) Stress

**DOI:** 10.3390/plants12010115

**Published:** 2022-12-26

**Authors:** Rafia Urooj Saman, Muhammad Shahbaz, Muhammad Faisal Maqsood, Nian Lili, Usman Zulfiqar, Fasih Ullah Haider, Nargis Naz, Babar Shahzad

**Affiliations:** 1 Department of Botany, University of Agriculture, Faisalabad 38040, Pakistan; 2 Department of Botany, The Islamia University of Bahawalpur, Bahawalpur 63100, Pakistan; 3 College of Forestry, Gansu Agricultural University, Lanzhou 730070, China; 4 Department of Agronomy, Faculty of Agriculture and Environment, The Islamia University of Bahawalpur, Bahawalpur 63100, Pakistan; 5 Key Laboratory of Vegetation Restoration and Management of Degraded Ecosystems, South China Botanical Garden, Chinese Academy of Sciences, Guangzhou 510650, China; 6 University of Chinese Academy of Sciences, Beijing 100039, China; 7 Tasmanian Institute of Agriculture, University of Tasmania, Hobart, TAS 7001, Australia

**Keywords:** plant growth, Brassica, EDTA, lead toxicity, ionic homeostasis, porphyrin ring, antioxidants

## Abstract

Lead (Pb) toxicity imposes several morphological and biochemical changes in plants grown in Pb-contaminated soils. Application of ethylenediamine tetraacetic acid (EDTA) in mitigating heavy metal stress has already been studied. However, the role of EDTA in mitigating heavy metal stress, especially in oilseed crops, is less known. Therefore, the study aimed to explore the potential effect of foliar application of 2.5 mM EDTA on two different varieties of *Brassica juncea* L., i.e., Faisal (V1) and Rohi (V2), with and without 0.5 mM Lead acetate [Pb(C_2_H_3_O_2_)_2_] treatment. Statistical analysis revealed that Pb stress was harmful to the plant. It caused a considerable decrease in the overall biomass (56.2%), shoot and root length (21%), yield attributes (20.16%), chlorophyll content (35.3%), total soluble proteins (12.9%), and calcium (61.7%) and potassium (40.9%) content of the plants as compared to the control plants. However, the foliar application of EDTA alleviated the adverse effects of Pb in both varieties. EDTA application improved the morphological attributes (67%), yield (29%), and photosynthetic pigments (80%). Positive variations in the antioxidant activity, ROS, and contents of total free amino acid, anthocyanin, flavonoids, and ascorbic acid, even under Pb stress, were prominent. EDTA application further improved their presence in the brown mustard verifying it as a more stress-resistant plant. It was deduced that the application of EDTA had significantly redeemed the adverse effects of Pb, leaving room for further experimentation to avoid Pb toxification in the mustard oil and the food chain.

## 1. Introduction

Heavy metals are non-biodegradable trace elements and are considered harmful to plants, animals, and humans [1]. Some heavy metals are categorized as essential heavy metals, which are somehow required for plant growth [2], while others are classified as non-essential heavy metals, which are not required for plant growth [3,4]. Lead (Pb) is a non-essential bluish-grey heavy metal present on the earth’s surface. Pb is found in various forms; lead acetate is highly soluble and more poisonous than lead oxide [5].

Exclusive peculiarities of Pb, e.g., solubilization, ductileness, and resistance to degradation led to its extravagant use in various products, industries, and chemical reactions [6]. Pb accumulates in the soil due to anthropogenic activities such as various metallurgical reactions, mining, etc., and natural phenomena such as land erosion, wildfires, and weathering [7]. It is considered as one of the dangerous soil contaminants that affects plant growth negatively [8] and diminishes the activities of various soil bacteria [9]. Pb in the soil accumulates in roots at cation exchange sites on the cell wall and ultimately enters the food-chain [10]. To minimize the toxic metals from the food-chain, contaminated soils are subjected to different biological, chemical, and physical techniques [11]. Among biological techniques, phytoremediation is an eco-friendly technique [12] that involves using plants to remediate soil contaminated with different metals [13,14].

A plant of agricultural importance, *Brassica juncea* L., belonging to the family Brassicaceae, is categorized among suitable plants for phytoremediation. The plant is also used as a vegetable, forage crop, in various medicines, and to extract edible oil from its seeds [15]. The oil obtained from the plant is highly valued for its flavor, aroma, food preservation, hair oil, and skin tonics [16]. Although Pb has deficient mobility in soil, it accumulates in the roots of the mustard plant. This accumulation results in high biomass production in a short growth period [17].

Various researchers have used different chelators to aid phytoremediation. Ethylenediamine tetra acetic acid (EDTA) is attracting the attention of many as a low-cost and highly effective chelating agent to remove various heavy metals from soil and water [18]. EDTA is known to aid heavy metal accumulation in different plant tissues. This can be helpful in eliminating harmful metals from contaminated soils [19]. EDTA, being a chelator, removes the toxic microbes and metals present in the soils or make them unavailable to the plants [20]. The positive role of EDTA in minimizing cadmium (Cd) accumulation in *Pelargonium* spp. was observed in plants grown under Cd-contaminated soils [21]. Similarly, Abbaszadeh-Dahaji et al. [22] showed that EDTA enhanced the phytoremediation efficiency of maize (*Zea mays*), sunflower (*Helianthus annuus*), and pumpkin (*Cucurbita pepo*) when grown in copper (Cu)-polluted soil. Tipu et al. [23] also verified the increase in uptake of 4.9 times in shoots and 2.6 times in roots by maize under EDTA treatment compared to the plants with no EDTA. Application of EDTA has shown to enhance zinc (Zn) accumulation in *Brassica* species [24].

EDTA application has been shown to improve antioxidant activities [25] and metal absorption by the plant roots [26]. Recent experiments have shown positive effects of EDTA on the *Brassica* species against different heavy metals, but the impact on Pb is least studied in a sand medium. Also, the Pb, one of the most immobile metals in soil, needs a chelator to transport to the plants. Brown mustard is an economically important crop, and extensive research is inevitable to culminate or at least minimize the annual yield losses due to heavy metal stress. To our knowledge, no study has been conducted in which EDTA was used as a foliar spray to mitigate the Pb toxicity in sandy soil conditions. In view of these facts, the current study was expected to alleviate the Pb-toxicity in mustard crops. This experimental study hypothesized that EDTA as a foliar treatment could improve plants’ growth, photosynthetic activity, and yield. Therefore, the current findings aimed to study the role of foliar application of EDTA on various morphological, physiological, and biochemical attributes of *Brassica*, both alone and in combination with Pb at various concentrations. Different yield-related characteristics were also investigated under these treatments.

## 2. Results

### 2.1. Morphological Attributes

**Figure 1:**
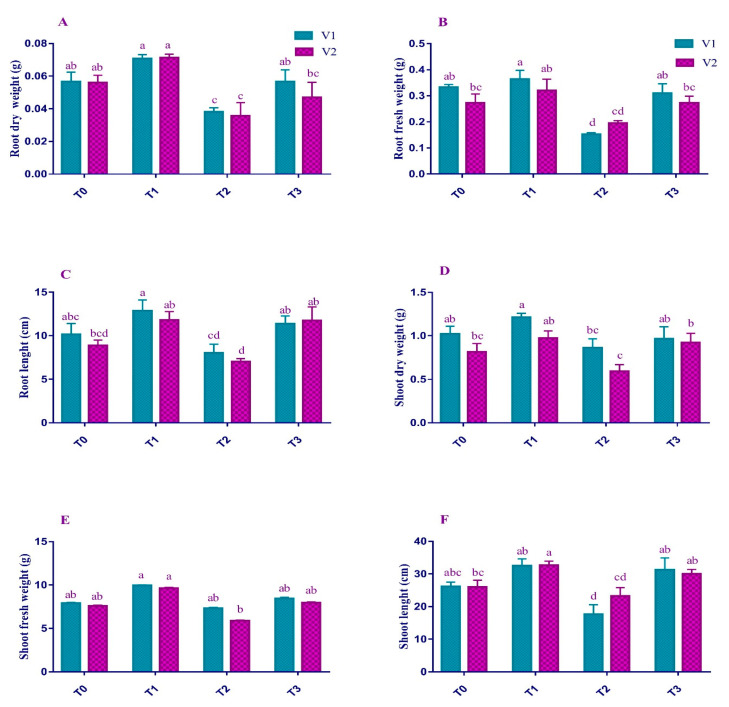
Effect of Pb and EDTA on (**A**) root dry weight (g), (**B**) root fresh weight (g), (**C**) root length (cm), (**D**) shoot dry weight (g), (**E**) shoot fresh weight (g) and (**F**) shoot length (cm) of brown mustard. Error bars above indicate the ± SE of three replicates. Means sharing the same letter for a parameter do not differ significantly at *p* ≤ 0.05. V1 = Faisal cultivar; V2 = Rohi cultivar; T_0_ = 0 mM EDTA + 0 mM Lead acetate; T_1_ = 2.5 mM EDTA + 0 mM Lead acetate; T_2_ = 0 mM EDTA + 0.5 mM Lead acetate; T_3_ = 2.5 mM EDTA + 0.5 mM Lead acetate.

The length of the shoot and root and their fresh and dry weights of both varieties (V1 and V2) were significantly (*p* < 0.01) affected due to Pb stress (Figure 1). There was a significant reduction in shoot length (32% and 10%), fresh weight (7% and 22%), and dry weight (15% and 27%) for V1 and V2, respectively, under T_2_. Similarly, a decrease due to Pb stress was observed in the root length (21% and 20%), fresh weight (54% and 28%), and dry weight (32% and 36%) for V1 and V2, respectively, as compared to the T_0_.

In addition, the foliar application of 2.5 mM EDTA compensated for the Pb-induced losses in shoot length (77% and 29%) and root length (42% and 67%) of both varieties, respectively. Maximum shoot fresh weight (35%) was observed in V2, fresh root weight (103%) in V1, shoot dry weight (56%) in V2, and root dry weight (49%) in V1 were recorded.

### 2.2. Photosynthetic Pigments

**Figure 2:**
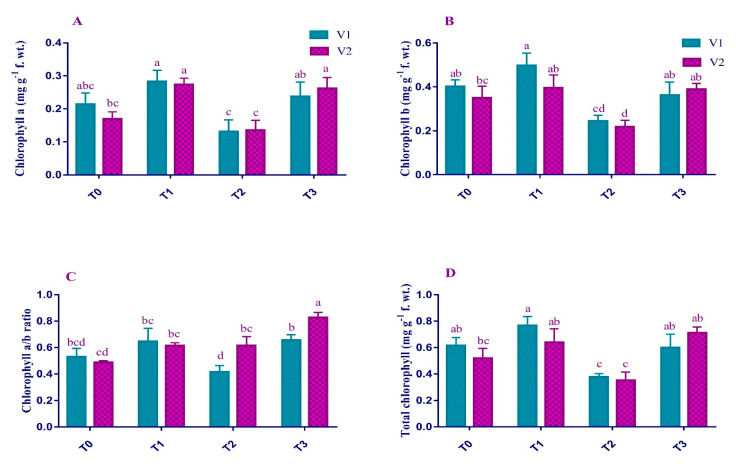
Effect of Pb and EDTA on (**A**) chlorophyll *a* (mg g^−1^ f. wt.), (**B**) chlorophyll *b* (mg g^−1^ f. wt.), (**C**) chlorophyll *a*/*b* ratio and (**D**) total chlorophyll (mg g^−1^ f. wt.) of brown mustard. Error bars above means indicate the ± SE of three replicates. Means sharing a letter for a parameter do not differ significantly at *p* ≤ 0.05. V1 = Faisal cultivar; V2 = Rohi cultivar; T_0_ = 0 mM EDTA + 0 mM Lead acetate; T_1_ = 2.5 mM EDTA + 0 mM Lead acetate; T_2_ = 0 mM EDTA + 0.5 mM Lead acetate; T_3_ = 2.5 mM EDTA + 0.5 mM Lead acetate.

Lead stress (Pb) and its interaction with Varieties (Pb x V) showed a significant (*p* < 0.05) trend for overall chlorophyll content (Figure 2). Recorded data for V1 revealed the decrease in chlorophyll a (38%), chlorophyll b (39%), total chlorophyll (77%), and carotenoids (23%) due to Pb stress (T_2_); whereas the data for V2 also showed a reduction in Chl. a (20%), Chl. b (37%), total Chl. (57%), and carotenoids (42%) under T_2_ as compared to T_0_. With the exogenous application of EDTA, there was a dramatic increase in the contents of Chl. a (80% and 93%), Chl. b (48% and 78%), total chlorophyll content (128% and 171%), and carotenoids (70% and 85%) of V1 and V2, respectively, as compared to the plants treated with only 0.5 mM Pb.

### 2.3. ROS and Enzymatic Antioxidants

**Figure 3:**
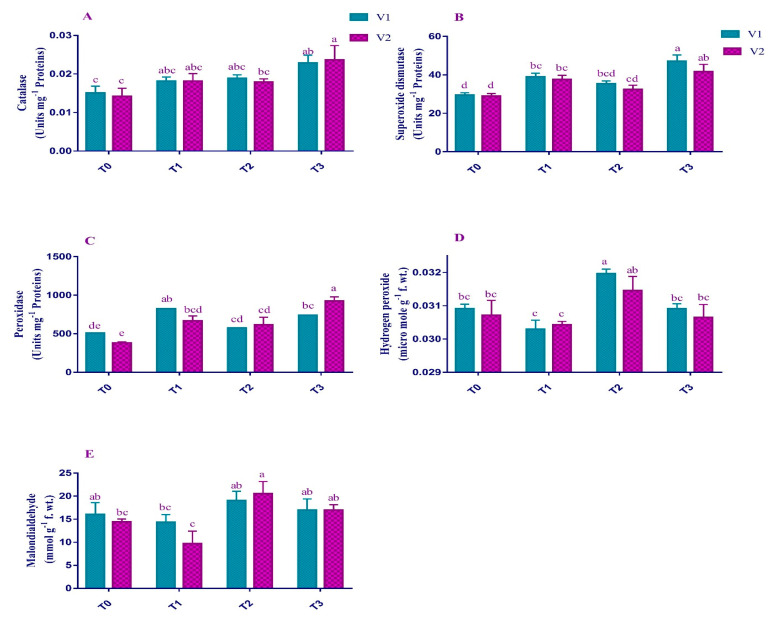
Effect of Pb and EDTA on (**A**) catalase (Units mg^−1^ proteins), (**B**) superoxide dismutase (Units mg^−1^ proteins), (**C**) peroxidase (Units mg^−1^ proteins), (**D**) hydrogen peroxide (µmol g^−1^ f. wt.), and (**E**) malondialdehyde (mmol g^−1^ f. wt.) of brown mustard. Error bars above means indicate the ± SE of three replicates. Means sharing a letter for a parameter do not differ significantly at *p* ≤ 0.05. V1 = Faisal cultivar; V2 = Rohi cultivar; T_0_ = 0 mM EDTA + 0 mM Lead acetate; T_1_ = 2.5 mM EDTA + 0 mM Lead acetate; T_2_ = 0 mM EDTA + 0.5 mM Lead acetate; T_3_ = 2.5 mM EDTA + 0.5 mM Lead acetate.

Exposing plants to Pb caused a highly significant (*p* < 0.001) effect on the production of H_2_O_2_ and MDA (Figure 3). V1 exhibited (3.38%) slightly more H_2_O_2_ content as compared to V2 (2.41%) under stress conditions. MDA content in V1 increased by 18% and 42% in V2 in Pb-stressed plants compared to control plants. However, the exogenous application of EDTA application somehow reduced the H_2_O_2_ content to 3.28% and 2.58% in V1 and V2, respectively, compared to T2. Similarly, EDTA application (T_3_) decreased the MDA contents to 10.9% and 17.3% in V1 and V2, respectively, compared to T_2_. Pb in interaction with varieties (Pb × V) showed a highly significant (*p* < 0.01) behavior for POD. Data collected showed that the activities of SOD, POD, and CAT in Pb-stressed plants were increased in both varieties except for CAT activities in V2 (Figure 3). A maximum increase in activities of SOD was observed in V1 (20%) and of POD in V2 (60%) under T_2_. In contrast, CAT activities increased by 25.5% in V1 and decreased by 25.7% in V2 under Pb stress conditions. However, antioxidant activities in both V1 and V2 were further increased by 33% and 28% for SOD, 28% and 50% for POD, and 20% and 32% for CAT, respectively, under EDTA application compared to Pb stress.

### 2.4. Non-Enzymatic Antioxidants

**Figure 4:**
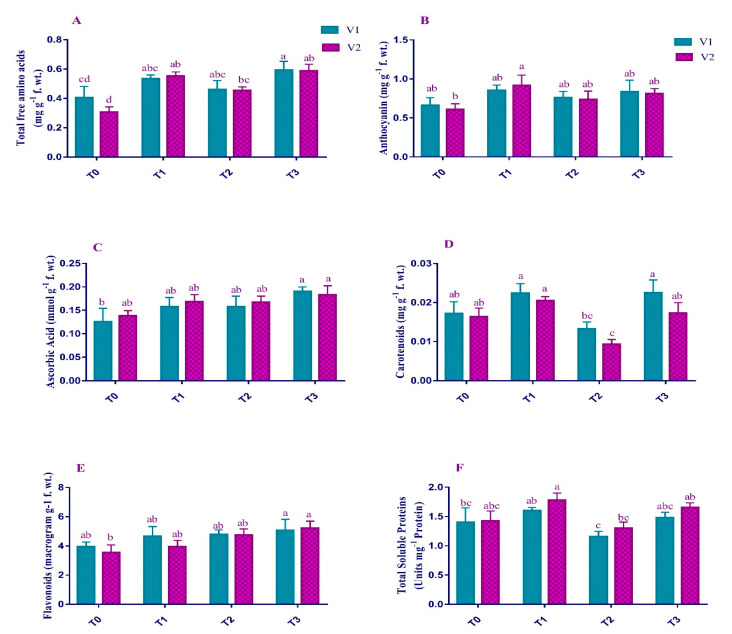
Effect of Pb and EDTA on (**A**) total free amino acids (mg g^−1^ f. wt.), (**B**) anthocyanin (mg g^−1^ f. wt.), (**C**) ascorbic acid (mmol g^−1^ f. wt.), (**D**) carotenoids (mg g^−1^ f. wt.), (**E**) flavonoids (µg g^−1^ f. wt.) and (**F**) total soluble proteins (Units mg^−1^ Proteins) of brown mustard. Error bars above means indicate the ± SE of three replicates. Means sharing a letter for a parameter do not differ significantly at *p* ≤ 0.05. V1 = Faisal cultivar; V2 = Rohi cultivar; T_0_ = 0 mM EDTA + 0 mM Lead acetate; T_1_ = 2.5 mM EDTA + 0 mM Lead acetate; T_2_ = 0 mM EDTA + 0.5 mM Lead acetate; T_3_ = 2.5 mM EDTA + 0.5 mM Lead acetate.

Non-Enzymatic antioxidants for the V1 and V2 showed greater significant (*p* < 0.01) behavior under Pb stress conditions (Figure 4). There was an increase in the contents of total free amino acid (13% and 48%), anthocyanin (14% and 21%), flavonoids (21% and 33%), and ascorbic acid (25% and 21%) in the stress conditions compared to the T0. On the other hand, Pb decreased the Total Soluble Proteins content in V1 (17%) and V2 (8%) as compared to the plants receiving no treatment. However, foliar application of EDTA retrieved the maximum losses of Total Soluble Proteins due to lead toxicity in both V1 (27.8%) and V2 (26.9%). In contrast, other antioxidants were further increased under EDTA application compared to T_2_. A maximum increase was observed in the Total amino acid content (29.42%) of V2, and the minimum increase was recorded in the flavonoid content (6.07%) of V1.

### 2.5. Mineral Ion Content

**Figure 5:**
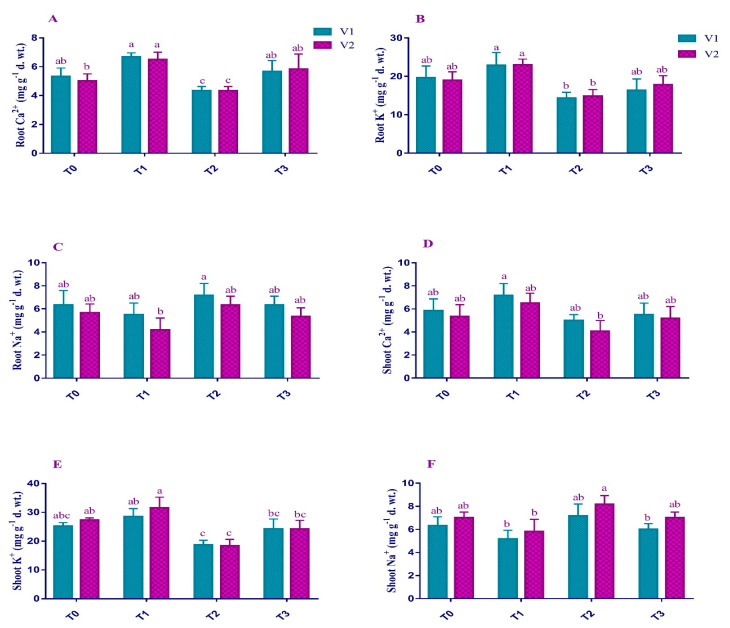
Effect of Pb and EDTA on (**A**) root calcium (mg g^−1^ d. wt.), (**B**) root potassium (mg g^−1^ d. wt.), (**C**) root sodium (mg g^−1^ d. wt.), (**D**) shoot calcium (mg g^−1^ d. wt.), (**E**) shoot potassium (mg g^−1^ d. wt.), and (**F**) shoot sodium (mg g^−1^ d. wt.) of brown mustard. Error bars above means indicate the ± SE of three replicates. Means sharing a letter for a parameter do not differ significantly at *p* ≤ 0.05. V1 = Faisal cultivar; V2 = Rohi cultivar; T_0_ = 0 mM EDTA + 0 mM Lead acetate; T_1_ = 2.5 mM EDTA + 0 mM Lead acetate; T_2_ = 0 mM EDTA + 0.5 mM Lead acetate; T_3_ = 2.5 mM EDTA + 0.5 mM Lead acetate.

Pb stress increased the Na^+^ contents in shoots (13% and 16%) and roots (13% and 11%) in V1 and V2, respectively, compared to the non-stressed plants (Figure 5). The maximum Na^+^ content (8 mg/g) was observed in shoots of V2 under Pb stress. Similarly, the Ca^2+^ concentration was decreased in shoots (14% and 23%) and roots (14% and 9%) of V1 and V2, respectively, under T_2_. Similarly, shoots (25% and 32%) and roots (27% and 21%) of V1 and V2, respectively, exhibited a decrease in K^+^ concentration. Maximum Ca^2+^ (5 mg/g) and K^+^ (18 mg/g) were observed in shoots of V1. When the foliar application of EDTA was done, the Na^+^ content in the shoots (16% and 14%) and roots (11% and 15%) decreased compared to the stressed plants. The concentration of Ca^2+^ was increased in shoots (10% and 27%) and roots (17% and 20%) of both V1 and V2, respectively, under T_3_. K^+^ concentration also showed a similar trend of increase in the shoot (29% and 31%) and roots (13% and 20%) of both V1 and V2, respectively, under T_3_ as compared to T_2_.

### 2.6. Yield Attributes

**Figure 6:**
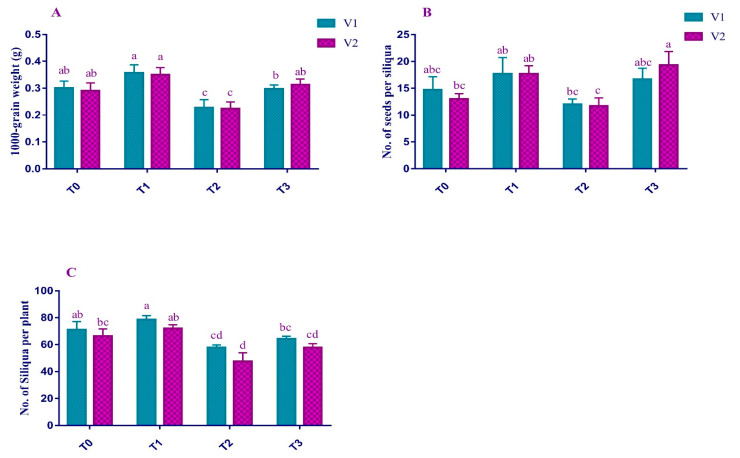
Effect of Pb and EDTA on (**A**) 1000 seed weight (g), (**B**) number of seeds per siliqua and (**C**) number of siliqua per plant, and of brown mustard. Error bars above means indicate the ± SE of three replicates. Means sharing a letter for a parameter do not differ significantly at *p* ≤ 0.05. V1 = Faisal cultivar; V2 = Rohi cultivar; T_0_ = 0 mM EDTA + 0 mM Lead acetate; T_1_ = 2.5 mM EDTA + 0 mM Lead acetate; T_2_ = 0 mM EDTA + 0.5 mM Lead acetate; T_3_ = 2.5 mM EDTA + 0.5 mM Lead acetate.

Different Yield parameters (No. of Siliquae per plant, No. of seeds per Siliqua, and 1000-seeds weight) were recorded to analyze the individual and cumulative effects of Pb and EDTA on V1 and V2 (Figure 6). Analysis revealed a decrease in the No. of Siliquae per plant (18% and 28%), No. of seeds per Siliqua (18% and 10%), and 1000-seeds weight (24% and 21%) in V1 and V2, respectively, consequently to Pb stress. Comparatively, the EDTA application resulted in maximum compensation of the yield losses due to Pb. All yield parameters, i.e., No. of Siliquae per plant (11% and 21%), No. of seeds per Siliqua (38% and 65%), and 1000-seeds weight (17% and 21%) were improved under EDTA treatment as compared to the Pb-stressed plants.

### 2.7. Principal Components Analysis (PCA) and Heatmap Analysis

**Figure 7:**
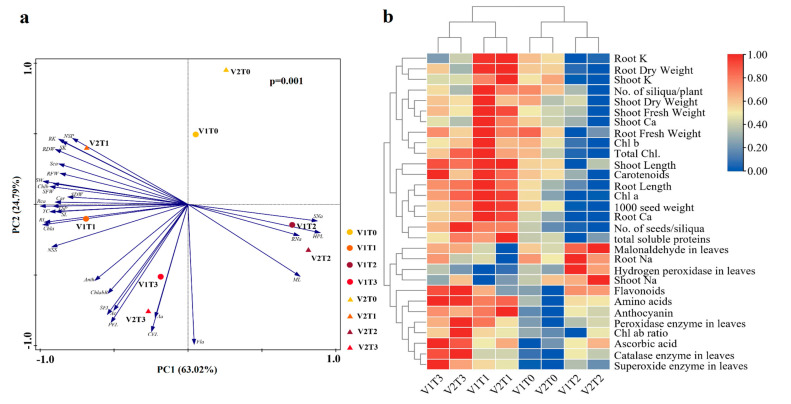
(**a**) Principal components analysis (PCA) and (**b**) heatmap analysis biplot between various attributes of brown mustard revealed the impact of foliar application of ethylenediamine tetraacetic acid (EDTA) under lead (Pb) stress on physiological and biochemical aspects. V1 = Faisal cultivar; V2 = Rohi cultivar; T_0_ = 0 mM EDTA+ 0 mM Lead acetate; T_1_ = 2.5 mM EDTA + 0 mM Lead acetate; T_2_ = 0 mM EDTA + 0.5 mM Lead acetate; T_3_ = 2.5 mM EDTA + 0.5 mM Lead acetate. RFW, Root Fresh Weight; RDW, Root Dry Weight; RL, Root Length; SFW, Shoot Fresh Weight; SDW, Shoot Dry Weight; SL, Shoot Length; CEL, Catalase enzyme in leaves; SEL, Superoxide enzyme in leaves; PEL, Peroxidase enzyme in leaves; HPL, Hydrogen peroxidase in leaves; ML, Malonaldehyde in leaves; ChlabR, Chl ab ratio; TC, Total Chl; SW, 1000 seed weight; NSS, No. of seeds/siliqua; NSP, No. of siliqua/plant; Rca, Root Ca; RK, Root K; RNa, Root Na; Sca, Shoot Ca; SK, Shoot K; SNa, Shoot Na; Ac, Amino acids; Anth, Anthocyanin; Aa, Ascorbic acid; Car, Carotenoids; Fla, Flavonoids; TSP, total soluble proteins.

As a result of the PCA, 87.81% of the total variation was explained by the first two principal components (63.02% and 24.79%, respectively; Figure 7a). The position of each variable in the loading plot (Figure 7) described its relationship to the other variables. Accordingly, the V2T1 treatment was distributed in the first quadrant, V1T0 and V2T0 treatments in the second, V1T2 and V2T2 treatments in the third, and V1T1, V1T3, and V2T3 treatments in the fourth quadrant, and was derived by an ANOSIM test (*p* < 0.05), so there were significant differences between treatments. Each plant characteristic was represented as a vector, and the angles between them correspond to the degree of correlation. Orthogonal variables (and components) were completely uncorrelated; variables with an angle of 180° are entirely, but negatively, correlated. The plots showed no clear pattern, and the principal components were strongly correlated with multiple plants characteristics.

To study the changes in individual plant characteristics under different treatments, the results of all plant characteristics measured under the eight treatments were normalized and then plotted in a cluster heat map (Figure 7b). The results show that the eight treatments can be divided into two categories, with V1T3, V2T3, V1T1, and V2T1 clustered into one category. Under these four treatments, all indicators showed high measurements. Correspondingly, V1T0, V2T0, V1T2, and V2T2 clustered into another category. In contrast to the results of the previous category, all indicators showed lower results under this category.

## 3. Discussion

Current studies support our hypothesis that the foliar application of EDTA has ameliorated the physical, physiological, and biochemical attributes due to toxicity caused by lead acetate. The study included two *Brassica juncea* L. cultivars, Faisal (V1) and Rohi (V2), being treated with 0 mM and 0.5 mM lead acetate with and without a foliar spray of 2.5 mM EDTA.

It was found in current studies that lead acetate caused a considerable reduction in morphological attributes such as length and fresh and dry weights of plant roots and shoots (Figure 1). This decrease may be due to the toxic effects of Pb exposure. Plants become unable to absorb nutrients or perform their metabolism normally and ultimately reduced growth [27,28]. Pb also disturbs the uptake of various minerals leading to the retardation of cell development and elongation [29]. However, the EDTA application improved the growth parameters of both V1 and V2. EDTA is known to be the best chelator [30]. Thus, it considerably improves the mineral’s phyto-availability in the soil and aids in the absorption and translocation of those minerals [31].

It has been investigated in the current studies that lead toxicity reduced the physiological activity of the plant to a severe concern. There was a noticeable reduction in the photosynthetic pigments of V1 and V2 under T_2_ (Figure 2). This decline in chlorophyll content because of Pb stress ultimately inhibits photosynthetic activities [32]. It was suggested that Pb might add iron (Fe) ions in place of magnesium (Mg) in the porphyrin ring of chlorophyll and eventually slow down the photosynthetic process [33,34]. However, the exogenous application of EDTA, in current studies, played a protective role in maintaining the chlorophyll content on a significant level. This might be due to improved activities of the electron transport chain and other protein complexes, ultimately enhancing the photosynthetic ability [35].

Reactive oxygen species (ROS) act as messengers in plants reporting stress elevation [36]. Heavy metals (Cu > Zn > Cd > Pb) cause an increase in ROS production in plant cells [37]. Similarly, our study also showed a higher production rate of H_2_O_2_ and MDA under T_2_. Higher dosages of heavy metals such as Cd might lead to the synthesis of ROS like H_2_O_2_, a typical response in Brassica spp. under stress conditions [38]. In current studies, foliar application of EDTA reversed the ROS production and thus played a key role against Pb stress (Figure 3). This might be due to the chelating properties of EDTA at the cellular level, which increase the antioxidant activities of the plants under stress conditions [39].

In both V1 and V2, the POD, SOD and CAT activity showed significantly different responses in control and stress-treated plants (Figure 3). The activities of all the enzymes increased under Pb stress. Under heavy metals such as Pb and Cd, *Zea mays* raised their antioxidant activities as a critical response [40]. Conversely, for current studies, EDTA application as a foliar spray increased antioxidant enzymes’ activity in both varieties. The most plausible reason for this increase is the decrease in oxidative pressure under EDTA treatment. It increases the antioxidant activity, thus rendering the *Brassica napus* to combat ROS [41].

It was observed that the total soluble protein (TSP) content was reduced under Pb stress in both varieties. It might be due to the dysfunction of the photosynthetic apparatus under Pb stress, resulting in reduced protein content [42]. In T_3_, EDTA compensated for the loss of soluble proteins up to a considerable level. This effect is related to the fact that EDTA may increase protein levels by controlling transcription and translation factors, and the structural pattern of proteins and enzymes [43].

In the present study, the application of Pb increased anthocyanins compared to the plants under control conditions (Figure 4). Under stress conditions, plants defend themselves by enhancing the production of anthocyanins because of their antioxidant activities and regulating the osmotic balance in the plants [44]. EDTA further enhances the leaves’ anthocyanin ratio, indicating more antioxidant potential. Our findings were very similar to Mousavi et al. [45]. High anthocyanin under EDTA application signifies more protection against ROS [46].

Total free amino acid content amplified in V1 and V2 because of Pb stress conditions. Improvement in the synthesis of some amino acids is owed to the decrease in protein synthesis [47]. Abiotic stress conditions produce some stress proteins that might ultimately reduce the amino acid concentration in certain plants [48,49]. Foliar application of EDTA induced the amino acid content in the current studies. These findings were identical with the findings of Nawaz et al. [50], who demonstrated the amino acid content in Brassica napus under EDTA.

Pb toxicity caused an increase in the flavonoid content in both varieties as compared to the control. According to Giannakoula et al. [51], this increase might be due to a decrease in Chl. *a* content of the plants under toxic metals, thus increasing the plant’s stress tolerance; EDTA application showed a more positive effect on flavonoid content among Pb-stressed plants. This suggests that *Brassica juncea* L. is one of the suitable plants for phytoremediation, as plants with higher flavonoid contents under heavy metal, such as Cu, stress are categorized as so [52].

The present study documented a considerable increase in ascorbic acid (AsA) content under Pb stress (Figure 4) These results suggest that the higher level of AsA in various plants might be the reason for its relative higher tolerance to Pb-stress [53]. On the other hand, EDTA application had no significant effect on the AsA content of the plant. Similar findings were achieved by Tammam et al. [54] under EDTA application with Pb stress on a different plant.

Mineral ions (Na^+^, Ca^2+^, K^+^) responded differently to Pb toxicity (Figure 5). Pb enhanced the Na uptake and reduced both varieties’ Ca and K uptake. Na imbalance could be justified as Pb accumulation influences the distribution of various ions and nutrients, ultimately compromising plant growth [55]. Ca decrease is attributed to the impairment of the calcium gates under Pb stress or may be due to more lead affinity on calcium transporting channels [56]. Pb blocks the passages for many ions and hinders their absorption from the soil into roots [57].

**Figure 8:**
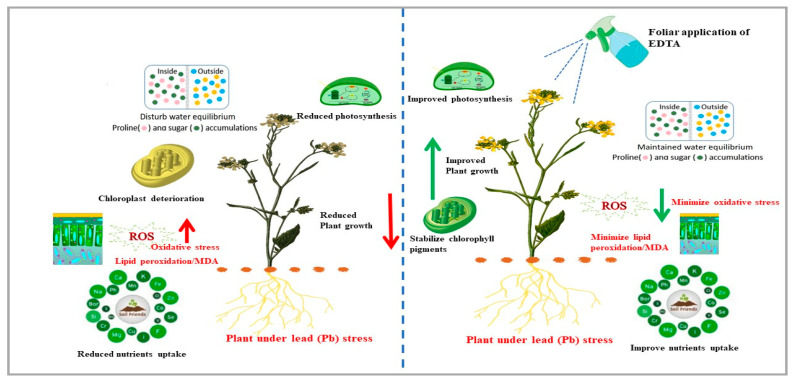
A theoretical scheme represents two different mechanisms of brown mustard plants grown under lead (Pb) contaminated soil with and without foliar application of ethylenediamine tetraacetic acid (EDTA). Pb stress adversely affects the morphological, biological, and physiological characteristics of the brown mustard plant, resulting in minimizing the uptake of the essential nutrients, disturbing water equilibrium, enhancing the oxidative stress, and deteriorating the organelles’ structures, i.e., mitochondria and chloroplast, which ultimately reduced the photosynthesis activity and plants growth. The plant cells are not adequately protected by this stress-responsive mechanism, which causes excessive MDA release from reactive oxygen species-damaged cells. The situation can be the exact opposite in the case of brown mustard plants whose leaves have been exposed to the foliar application of EDTA. EDTA induced stomatal closure to prevent evapotranspiration and mediate stress-responsive pathways to lessen cell damage during Pb stress, which helps to enhance and stabilize the chlorophyll pigments and osmolytes accumulation, which results in improved photosynthesis activity, reduced oxidative stress, and ultimately improved the yield of the plant.

Current studies focused on the number of siliquae per plant, seeds per siliqua, and 1000 seeds-weight as the yield parameters of Pb-affected plants (Figure 6). The overall yield of the plants was antagonistically affected by the Pb application. This severe yield drop is due to the collective effects of a decrease in these individual parameters [58]. It is also reported that the reduction of yield-measuring factors is the reason for overall yield reduction under Pb toxicity [59]. Additionally, exogenous administrations of EDTA were successful in enhancing the properties of leaf gas exchange and osmolytes accumulation as well as stabilizing chlorophyll pigments (Figure 8). These systems are crucial for maintaining brown mustard growth when soil is contaminated with Pb. Although, EDTA compensated the losses of yield under Pb stress but failed to limit the entry of Pb into plants and ultimately in the mustard oil and food. Increasing demand and consumption of mustard oil appeal to further studies and devise better strategies to limit the entry of Pb into the food chain under field conditions.

## 4. Materials and Methods

### 4.1. Chemicals

Chemicals such as Ethylenediamine tetraacetic acid (EDTA), lead acetate, trichloro-acetic acid (TCA), 80% acetone solution, potassium iodide, thiobarbituric Acid (TBA), and phosphate buffers were purchased and provided by the Department of Botany, University pf Agriculture, Faisalabad, Pakistan.

### 4.2. Experimental Design and Setup

A pot experiment was conducted during the last week of November 2021 to study the effects of Lead acetate on two different varieties of *Brassica juncea* L., namely Faisal (V1) and Rohi (V2), with and without the application of EDTA. Seeds were collected from the Ayub Agricultural Research Institute (AARI), Faisalabad, Pakistan. The experiment was executed at the wire house of Old Botanical Garden at the University of Agriculture Faisalabad, Pakistan (Latitude: 31°25′46.8048″. Longitude: 73°4′14.3112″). An amount of 5 kg of sand washed with tap water and then dried sand was filled in each pot having dimensions, i.e., 25 cm × 22.5 cm × 20 cm.

Hoagland’s solution and other treatment solutions were prepared in the laboratory of the Department of Botany, University of Agriculture Faisalabad. Hoagland’s nutrient solution contained K(NO_3_)_2_ 3 mM; Ca(NO_3_)_2_ 2 mM; KH_2_(PO_4_) 0.1 mM; MgSO_4_ 1 mM; H_3_BO_3_ 0.05 mM; MnCl_2_ and FeNa-EDTA 0.012 mM. The experimental plan was comprised of a total of 24 pots (4 × 2 × 3) including 4 Pb and EDTA treatments, two varieties, having three replications, arranged by using completely randomized design (CRD). A total of 10 seeds of each variety were sown in each pot and were thinned 15 days after germination, rendering 5 plants in each pot. After 30 days of sowing, plants were exposed to lead acetate followed by foliar application of EDTA as per follows:

T_0_ (0 mM EDTA+ 0 mM Lead acetate)

T_1_ (2.5 mM EDTA+ 0 mM Lead acetate)

T_2_ (0 mM EDTA+ 0.5 mM Lead acetate)

T_3_ (2.5 mM EDTA+ 0.5 mM Lead acetate)

Plants were treated for 4 weeks, and the Hoagland solution was applied when and if needed to ensure proper growth. Weeds were removed from pots by hand.

### 4.3. Morphological Attributes

Two plants from each replicate were harvested in the midweek of February 2022, and immediately after collection, the fresh weight of the root and shoot was weighed with the help of electrical balance. Lengths (cm) of shoots and roots were measured with the help of measuring tape according to the procedure mentioned by [58]. Later, these shoots and roots were shifted to oven drying for three days at 60 °C. After complete drying, the dry weight of the shoots and roots was recorded with the help of balance. Dried samples were set aside for further analyses.

### 4.4. Photosynthetic Pigments

The method of Arnon [60] was followed to measure the photosynthetic pigments (Chl. a, Chl. b, and carotenoids). A total weight of 0.1 g of each fully grown leaf of brown mustard plant was added in 5 mL of 80% acetone solution. The samples were left in the dark for 10–12 h at room temperature. Afterwards, the absorbance of these samples was recorded at 663 nm, 645 nm, and 480 nm with the help of a spectrophotometer (IRMECO U2020, IRMECO Gmbh, Schwarzenbek, Germany).

### 4.5. Oxidants Activities in Plant Leaves

The Velikova et al. [61] method was applied to check the activity of H_2_O_2_. In short, 0.25 g of the full-grown leaf of brown mustard plants was taken and homogenized in 5 mL of prepared solution of 0.5% trichloro-acetic acid TCA. Centrifugation was done and 500 µL supernatant was added in 500 µL of phosphate buffer and 1ml of potassium iodide and were mixed with the help of a vortex. At 390 nm, absorbance was recorded carefully. The Cakmak and Harst [61] protocol for MDA calculation was followed. For this purpose, 0.3 g of leaf extract was homogenized with 3 mL of 0.5% TCA solution and subjected to centrifuge. In the test tube, 1000 µL of 0.5% thiobarbituric acid (TBA) and 1 mL of the supernatant was added and incubated for 15 min at 95 °C. The absorbance was then observed at 532 nm and 600 nm on an IRMECO Spectrophotometer.

### 4.6. Enzymatic Antioxidants Activities in Plants Leaves

The antioxidant activities of brown mustard were determined by taking 0.25 g of sample, then grinding in 5 mL of potassium phosphate buffer using a chilled pestle and mortar. Grinded mixtures were then centrifuged for 15 min at 12,000 rpm. After centrifugation, the supernatant was collected and preserved for further analysis. The method of Chance and Maehly [62] was adopted for the measurement of catalase activity. An amount of 1.9 mL chilled phosphate buffer was added in the cuvette in addition to 1 mL H_2_O_2_ and 1000 µL of supernatant to check the absorbance at 240 nm at 0 s, 30 s, 60 s, and 90 s via using a spectrophotometer. According to Spitz and Oberley [63], SOD activity was measured using a quartz cuvette in which needed solutions were added appropriately. First, 400 µL distilled water, followed by the addition of 250 µL potassium phosphate buffer, 100 µL of L-methionine solution, 100 µL Triton X solution, 50 µL of NBT Solution, 50 µL of supernatant and at the end, 50 µL of riboflavin added. Samples were placed under a lamp for 30 min to start the reaction. The absorbance of the samples was observed at 560 nm via using a spectrophotometer. The Chance and Maehly [64] method of measuring the POD activity was followed. In cuvette, 750 µL of phosphate buffer, 100 µL guaiacol, 50 µL of supernatant, and 100 µL of H_2_O_2_. The absorbance was recorded for 0 s, 30 s, 60 s, and 90 s at 470 nm via using a spectrophotometer.

### 4.7. Non-Enzymatic Antioxidants Activities of Plants

To determine the total soluble proteins, 0.25 g of each fresh leaf sample was grinded in 5 mL of Phosphate buffer and centrifuged at 12,000 rpm for 15 min. An amount of 5 mL of this supernatant was taken in the test tube with 5 mL of Bradford reagent. Vortex samples and absorbance were checked at 595 nm via using a spectrophotometer [65]. To determine the total free amino acids, 0.5 mL of 10% pyridine solution, 0.5 mL of 2% ninhydrin, and 0.5 mL of supernatant were added in test tubes. Samples were water bathed for 30 min at 50 °C and then diluted up to 25 mL. Then, the absorbance of these samples was checked at 570 nm via using a spectrophotometer [66]. The technique of Stark and Wray’s [67] was applied to estimate the level of anthocyanin. Briefly, 0.1 g of fresh leaf sample was crushed in 2 mL of acidified methanol and incubated at 90 °C for 60 min. After the incubation period, the absorbance of these samples was recorded on the spectrophotometer at 535 nm via using a spectrophotometer.

To determine Flavonoids, 100 mg of the leaf was soaked in 5 mL of 80% acetone solution for 10–12 h. Amounts of 1 mL of leaf extract,4 mL of distilled water, 600 µL of 5% sodium nitro oxide (NaNO_2_), 500 µL of 10% AlCl_3_, and 2 µL of 1M sodium hydroxide (NaOH) were added in test tubes. Then, the observations at 510 nm were recorded via a spectrophotometer [68]. Mukherjee and Choudhuri’s [69] method was applied to determine the ascorbic acid content. An amount of 5 mL of 6% TCA and 100 mg of the brown mustard leaf were ground and centrifuged for 5 min at 12,000 rpm. Amounts of 1 mL of the supernatant, 2 mL of 2% DNPH, and one drop of thiourea were mixed and subjected to a water bath at 50 °C for 20 min. After that, 2.5 mL of 80% H_2_SO_4_ was added to each sample to check the absorbance at 530 nm via using a spectrophotometer.

### 4.8. Ion Analysis (Na^+^, Ca^2+^, K^+^)

An amount of 0.1 g of oven-dried plant samples was added to the digestion flask with 2 mL H_2_SO_4_ and left in the dark for 10–12 h. After 10 h, flasks were placed on the hot plate, and H_2_O_2_ was added to the digestion flasks while the hot plate was on until the mixture appeared transparent or colorless. Samples were then filtered and diluted. The concentration of different ions (Na^+^, Ca^2+^, K^+^) in shoots and roots was determined by using a flame photometer (Sherwood flame Photometer-410, Sherwood Scientific Ltd. Cambridge, UK).

### 4.9. Yield Attributes

The number of siliqua per plant and seeds per silique were recorded from the freshly harvested plants from each pot. For measuring the weight of 1000 seeds, a hundred seeds from each treatment were sundried for a few days and then weighed on an electrical balance. The weight was then converted to weight per 1000 seeds [58].

### 4.10. Statistical Analysis

The statistical analyzation of the data recorded was carried out using statistical software (Statistix, Version 8.1) to test the significance of different treatments at the 5% level. Additionally, graphical work was done using Microsoft Excel (2019 version) (Microsoft Corporation, Redmond, WA, USA).

## 5. Conclusions

The present study establishes the changes in two different varieties (V1 and V2) of *Brassica juncea* L. exposed to Pb in combination with EDTA. Both varieties demonstrated much resistance to the Pb stress, owing to the foliar application of EDTA. Due to Pb stress, the growth, photosynthetic pigments, enzymatic characteristics, and yield of the plants were adversely affected. It was also recorded that Pb caused increased production of ROS, ultimately damaging the cell membranes. Due to stress, metal ions’ (Na^+^, Ca^2+^, and K^+^) uptake and translocation were also disturbed. However, treatment with a chelating agent, EDTA, distinctly indicated its positive role in recovering stress-induced losses. Foliar application of EDTA substantially reduced the partially oxidized harmful ions and thus proved to be a favorable chemical for plants or crops grown in Pb-contaminated areas. Overall, Faisal (V1) performed better and exhibited more resistance to Pb than V2. This study highlighted the promising effects of EDTA against Pb toxicity at smaller levels in pots-based experiments. However, EDTA is not easily degradable and thus can affect the phyto-availability of other micro and macro nutrients. Furthermore, investigations should be carried out with some other chelating agents to study the phytoremediation of toxic metals in field settings on a larger scale.

## Data Availability

Not applicable.
